# Bakuchiol perturbs purine metabolism and ABC transporter pathways in *Streptococcus agalactiae*, possibly affecting *S. agalactiae*-induced mastitis

**DOI:** 10.3389/fmicb.2026.1961106

**Published:** 2026-10-28

**Authors:** Rui Wang, Ya-Rui Jin, Wei-Qi Jiang, Jun-Xia An, Guo-Liang Mou, Li-Jing Zhang, Ying-Qian Liu, Zhi-Jun Zhang, Shao-Yong Zhang

**Affiliations:** 1 Key Laboratory of Biochemistry and Molecular Biology in Universities of Shandong Province, Weifang University, Weifang, China; 2 School of Pharmacy, Lanzhou University, Lanzhou, China; 3 Key Laboratory of Vector Biology and Pathogen Control of Zhejiang Province, College of Life Science, Huzhou University, Huzhou, China

**Keywords:** antimicrobial mechanism, bakuchiol, inflammatory factor, model of mouse mastitis, *Streptococcus agalactiae*, untargeted metabolomics analysis

## Abstract

**Objective:**

To evaluate the bacteriostatic effect of Bakuchiol (Bak) against *Streptococcus agalactiae*, explored its antibacterial mechanism *in vitro*, and investigated its *in-vivo* therapeutic activity in a mouse mastitis model.

**Methods:**

The Oxford cup assay was performed to test its inhibitory activity on *S. agalactiae*. Scanning electron microscopy and qPCR were employed to unravel the in-vitro molecular mechanism of Bak. Metabolomics was conducted to assess the impact of Bak on bacterial metabolites. A *S. agalactiae*-induced mouse mastitis model was constructed, and its therapeutic efficacy in mice was evaluated via histopathological sections and detection of inflammatory cytokine (IL-6, IL-8, IL-1β and TNF-α) expression.

**Results:**

Among 24 natural small-molecule phenolic compounds, Bak exhibited favorable antibacterial activity against *Streptococcus agalactiae*. Mechanistic studies revealed that Bak significantly inhibited the production of virulence factors and the expression of virulence genes. Notably, it suppressed biofilm formation by disrupting initial adhesion, eliminating mature biofilms, and reducing biofilm metabolism and extracellular polymer production. Untargeted metabolomics analysis revealed that Bak might produce antibacterial activity through significant disruption of purine metabolism and ABC transport-associated metabolic pathways in *S. agalactiae*. Acute toxicity studies indicated that Bak exhibited low toxicity. In a mouse mastitis model, Bak significantly improved mastitis by mitigating tissue damage, and downregulating pro-inflammatory cytokine levels.

**Conclusion:**

Bak attenuated *S. agalactiae* pathogenicity through a multi-target mechanism, positioning it as a potential therapeutic candidate for bacterial mastitis.

## Graphical abstract

**Figure:**
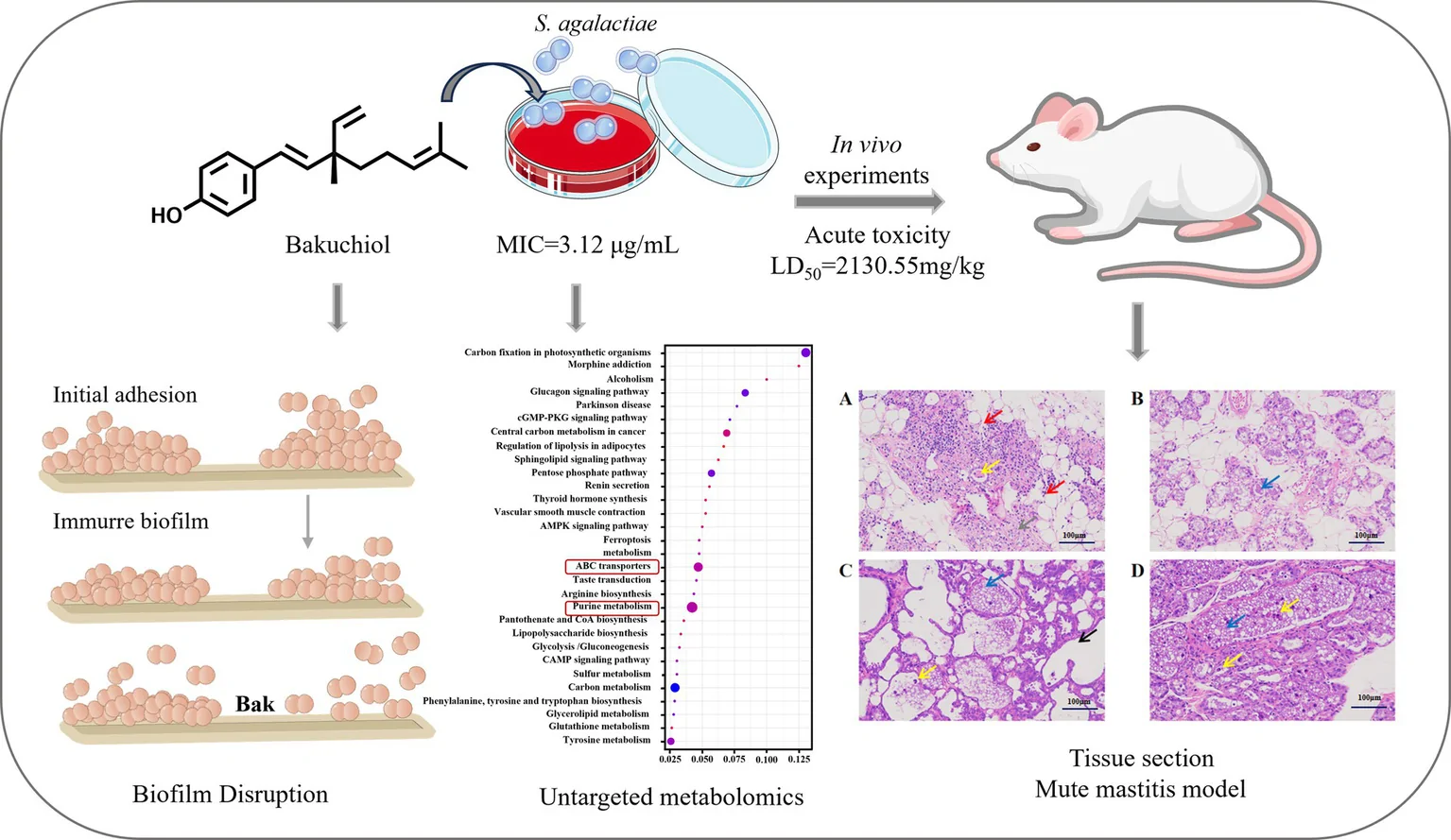

## 1 Introduction

Mastitis is one of the most common diseases affecting dairy cows. It primarily impacts animal welfare, but also hinders milk production quality, significantly affecting the profitability of dairy farms (Bisutti et al., 2023; Ruegg, 2017). Mastitis typically occurs due to the penetration of various microorganisms into the mammary gland. It manifests in two forms: the clinical form, characterized by evident inflammation, breast swelling, and changes in milk composition; and the subclinical form, where animals show no apparent signs of illness despite an ongoing inflammatory process or breast infection (Martin et al., 2018). Subclinical mastitis is the most challenging form, with an estimated incidence 15 to 40 times higher than its clinical counterpart (Bradley, 2002; Xiang et al., 2025; Xiang et al., 2026b). It represents a significant source of infection within herds, leading to substantial reductions in milk production (De Vliegher et al., 2018). Studies indicated *Streptococcus agalactiae* was a common pathogen in cows with subclinical mastitis, detected in 2.8% of 3,288 clinical mastitis samples (Gao et al., 2017; Zhang et al., 2018).

*Streptococcus agalactiae* (Group B Streptococcus, GBS) is a pathogenic microorganism that produces multiple virulence factors, such as promoting host cell adhesion and invasion, and evading the immune system (Xia et al., 2020; Zhai et al., 2025). These factors enable it to escape the host’s immune system and cause infection. *S. agalactiae* adheres to host cells through the interaction of bacterial adhesion factors with host cell surface receptors, thereby enhancing its ability to penetrate the host mucosal barrier and spread to other host tissues (Buscetta et al., 2014; Liu and Liu, 2022). Research indicated that lipoteichoic acid (LTA) and peptidoglycan within the cell wall of *S. agalactiae* could evade detection by the host immune system. Modifying LTA with D-alanine residues reduced the recognition of bacteria by host immune cells (Hernandez et al., 2021). Antibiotics represent the most direct and effective treatment for *S. agalactiae* infections. However, the extensive use of these drugs has led to their cumulative depletion, resulting in the alarming emergence and spread of antibiotic resistance (Leonard et al., 2018; J. Zhang et al., 2024). Consequently, the emergence and global dissemination of antimicrobial resistance (AMR) underscore the critical necessity of developing novel therapeutic strategies that transcend traditional bactericidal mechanisms.

Natural products and their derivatives represent valuable resources for discovering novel antimicrobial lead compounds (Karageorgis et al., 2021; Svendsen et al., 2025; van Santen et al., 2022). Phenolic compounds are widely distributed in plants and have been extensively studied for their remarkable antioxidant and antibacterial activities (Alu’datt et al., 2017; Jakobek and Blesso, 2024; Wang et al., 2023). Numerous phenolic compounds with antibacterial potential are abundant in various plant and food sources. Phenolic compounds in flaxseed oil could inhibit the growth of *Escherichia coli* and *Staphylococcus aureus* (Chen et al., 2025). The most extensively studied compounds include eugenol, carvacrol, thymol, resveratrol, cinnamaldehyde, and pyrogallic acid (Padilha da Silva et al., 2024). Overall, the primary mechanisms underlying the antibacterial activity of phenolic compounds involve their ability to disrupt bacterial cell membranes and inhibit key enzymes in bacterial metabolism (Sánchez-Arévalo et al., 2022; Sánchez-Arévalo et al., 2023; Tomczak and Gryta, 2023). However, the specific activity differences of phenolic compounds with varying structures against *S. agalactiae* and their mechanisms of virulence suppression remain unclear. To address this issue, we first conducted antimicrobial activity screening on 24 small-molecule phenolic compounds to identify effective lead compounds capable of inhibiting *S. agalactiae*.

In this study, we first screened 24 small-molecule phenolic compounds for anti-*S. agalactiae* activity, thereby identifying bakuchiol (Bak) as the most effective candidate drug. We then subjected Bak to a rigorous mechanistic interrogation, evaluating its effects on bacterial growth, key virulence factors (hemolysin, hyaluronidase), virulence gene expression, and multifaceted biofilm dynamics (formation, adhesion, pre-formed biofilm eradication, and hydrophobicity). Untargeted metabolomics analysis revealed that Bak might exert its antibacterial effects primarily through purine metabolism and ABC transporter pathways. Finally, the translational therapeutic potential of Bak was unequivocally demonstrated in a murine model of *S. agalactiae*-induced mastitis, assessing pathological damage and host inflammatory responses. Our work not only identified Bak as a promising natural anti-virulence agent but also provided a comprehensive pharmacological foundation for its development as a novel therapeutic strategy against *S. agalactiae* infections.

## 2 Experimental model and study participant details

### 2.1 *In vitro* antibacterial assay

The antibacterial activity of Bak against Gram-positive bacteria was determined using the microdilution broth method (Puente et al., 2025). *S. agalactiae* and *S. dysgalactiae* were cultured in BHI medium, while *E. coli* and *S. aureus* were cultured in MH medium. MIC was defined as the concentration at which the compound inhibited bacterial growth by at least 90% (MIC_90_).

$$\text{Corrected values}={\mathrm{OD}}_{\text{bacterial wilt}}-{\mathrm{OD}}_{\mathrm{no}\;\text{bacterial wilt}}$$

$$\text{Inhibition rate}\;(\%)=\frac{C-T}{C}\times 100\%$$

C: corrected turbidity values of the control.

T: corrected turbidity values treated with compounds.

### 2.2 Time growth curve

Bacterial suspensions in logarithmic growth phase (1 × 10^7^ CFU/mL) were incubated with different concentrations (1/2MIC, MIC, 2MIC) of Bak in 96-well microplates at 37 °C. BHI medium without compounds was used as a blank control group. The absorbance of the suspensions was measured at 600 nm using an enzyme meter at different incubation times (0, 2, 4, 6, 8, 10 and 12 h), and three sets of replicates were set for each concentration (Wang X. et al., 2022a).

### 2.3 Scanning electron microscope (SEM) observation

The bacterial solution at logarithmic growth stage was added into BHI medium containing Bak at MIC and incubated at 37 °C for 12 h with constant temperature and shaking, and BHI medium with equal amount of DMSO was used as a blank control. Collected bacterial cells precipitate, then added 2.5% glutaraldehyde fixative and fixed overnight at 4 °C. Fixed the treated bacterial pellet onto the sample stage surface. After gold spraying, observed and analyzed using scanning electron microscopy (SEM) (Liang et al., 2023).

### 2.4 Effect on *β*-hemolysin

#### 2.4.1 Preparation of bacterial suspension

1% (v/v) logarithmic growth phase bacterial solution was co-cultured with Bak at MIC concentration, and the blank control was the bacterial solution added with an equal amount of DMSO. After 12 h of incubation, the bacterial solution was washed three times and adjusted to an OD value of 0.8 at 620 mn, and then placed in a 4 °C environment for storage.

#### 2.4.2 Preparation of erythrocyte suspension

100 μL of sterilized defibrinated sheep blood was centrifuged at 3000 rpm for 5 min, and the supernatant was removed, and 100 μL of G-PBS solution (PBS with 0.1% glucose added) was added at a concentration of 0.2%.

#### 2.4.3 Determination of hemolytic inhibition

100 μL of the prepared bacterial suspension was added to each well of the first column of a 96-well plate and serially doubled with PBS. Each well was supplemented with 100 μL of erythrocyte suspension, and the reaction was carried out at 37 °C for 60 min, then transferred to a centrifuge tube and centrifuged at 3,000 rpm for 10 min. 100 μL of the supernatant was aspirated, and the absorbance value was measured at 540 nm and labeled as Ai. 0.1% SDS was used as positive control instead of bacterial suspension, and the absorbance was A0; the negative control was used as negative control with PBS instead of bacterial suspension, and the absorbance was A_0_’. The absorbance was measured as A_0_’. All experiments were set up with three parallel samples. The hemolysis rate (HR, %) was finally calculated by the following formula

$$\mathrm{HR}=\frac{A_{i}-A_{0}^{\prime }}{A_{0}-A_{0}^{\prime }}\times 100\%$$

The concentration of bacteria as the horizontal coordinate and hemolysis rate as the vertical coordinate, were plotted on a scatter plot and fitted with a Logistic equation to calculate the concentration of half-hemolysis rate bacterial fluid (EC_50_), and the hemolysis price (HT) and hemolysis inhibition rate (IR) were calculated according to the formula, respectively:

$$y=B_{2}+(B_{1}-B_{2})/(1+{\left(\frac{x}{x_{0}}\right)}^{p}$$

Where y is HR, x is concentration of bacterial fluid, B_1_, B_2_, x_0_ and *p* are constants.

$$\mathrm{HT}=10^{9}/{\mathrm{EC}}_{50}$$

$$\mathrm{IR}=(1-\frac{\mathrm{HT}}{{\mathrm{HT}}_{0}})\times 100\%$$

Where HT is the hemolytic price measured after compound action, and HT_0_ is the hemolytic price of the blank control (Xin et al., 2024).

### 2.5 Effect on hyaluronidase activity

#### 2.5.1 Preparation of hyaluronidase activity assay plate

Sodium hyaluronate and bovine serum albumin were prepared into 2 mg/mL and 50 mg/mL solutions with sterile PBS, respectively, and placed in a low temperature environment to dissolve. The prepared solution was added to the BHI semi-solid medium to final concentrations of 400 μg/mL and 10 mg/mL, respectively. Four Oxford cups were then placed on the medium to form round wells.

#### 2.5.2 Preparation of hyaluronidase standard

10 mg of standard enzyme was dissolved in 1 mL of sterile water and mixed thoroughly under ice bath conditions to make 10 mg/mL stock solution. Gradually dilute with sterile water to obtain a series of concentration solutions from 1829.50 to 28.59 U/mL, and freeze and store.

#### 2.5.3 Determination of hyaluronidase standard

10 μL of different concentrations of hyaluronidase standard solution were added into the wells, and three replicate wells were set up for each concentration, and 57.17 U/mL solution was used as the control. The reaction was incubated at 37 °C for 16 h. After the reaction was completed, 2 mol/L acetic acid solution was added to cover the whole surface. Twenty minutes later, the acetic acid solution was aspirated, and the diameter of the transparent zone was measured by vernier caliper. Take the logarithmic value of hyaluronidase standard activity as the horizontal coordinate, and the diameter of the transparent zone as the vertical coordinate to establish the standard curve equation.

#### 2.5.4 Determination of hyaluronidase activity

After 4 h of co-culture of logarithmic phase bacterial solution with different concentrations of Bak (control, 1/4MIC, 1/2MIC, MIC), 10 μL of the mixture was added to the plate wells, and three replicates were set for each treatment. The standard enzyme control was also set up, and all experiments were repeated three times, and the size of the clear zone was determined as described before. The difference between the measured control value and the theoretical value of the standard curve was used as the correction factor to correct the data of each experiment. The enzyme activity was calculated from the standard curve to obtain the hyaluronidase concentration (HTC_i_) of each treatment group, and the inhibition rate of enzyme activity (IRH) was calculated according to the formula using the bacterial solution without Bak as control (HTC_0_) (Wang et al., 2020)

$$\mathrm{IRH}=(1-\frac{{\mathrm{HTC}}_{i}}{{\mathrm{HTC}}_{0}})\times 100\%$$

### 2.6 Effects on the expression of virulence genes

Different concentrations of Bak (MIC, 1/2MIC) were co-cultured with the logarithmic growth phase of the bacterial suspension until overnight, and the bacterial suspension with equal amount of DMSO added was used as a blank control. After incubation, bacterial RNA was isolated, and cDNA was synthesized by reverse transcription. Six virulence genes were selected, and the prepared cDNAs were used as amplification templates to analyze the expression levels of the target genes in a relative quantitative manner. The relative expression of mRNA of each virulence gene was calculated using the 2^−△△Ct^ method with 16S as the internal reference gene, and the experiment was repeated three times (Priya et al., 2021).

### 2.7 Effects on the biofilms

#### 2.7.1 Inhibition assays of early adhesion

Graded concentrations of Bak (control, 1/2 MIC, MIC, and 2 MIC) and 1% (v/v) of the logarithmic growth phase of the bacterial fluids in BHI medium (containing 0.5% glucose, 3% NaCl) at 37 °C until the OD_600nm_ was about 0.5, followed by centrifugation at 5,000 rpm for 5 min, and the bacterial precipitates obtained were transferred to 96-wells of pre-added fibrinogen The obtained bacterial precipitate was transferred to 96-well culture plates with pre-added fibrinogen, incubated at 37 °C for 1 h, fixed with 25% formaldehyde solution, and then stained with crystal violet staining solution. Finally, the absorbance value was detected at 595 nm by an enzyme marker. The relative adhesion rate was calculated as follows (Xiang et al., 2026a; Xin et al., 2024).

$$\text{Relative adhesion rate}=(\mathrm{Bak}\;{\mathrm{OD}}_{595\;\mathrm{nm}})/(\mathrm{CK}\;{\mathrm{OD}}_{595\;\mathrm{nm}})\times 100\%$$

#### 2.7.2 Effect of initial biofilm formation on cell hydrophobicity

1% (v/v) logarithmic growth phase bacterial solution was inoculated in 3 mL BHI medium with Bak (control, 1/4MIC, 1/2MIC, MIC) and incubated at 37 °C for 12 h. After that, 500 μL of toluene was added to the test tubes, vortexed for 3 min, and then allowed to stand so that the two phases were separated. Finally, the lower aqueous phase was carefully aspirated and OD600 nm was measured before and after vortexing. The hydrophobicity inhibition percentage was calculated as follows (Kim et al., 2021).

$$\begin{array}{l} \text{Percent hydrophobicity}=\\ {}[\begin{array}{l} 1-({\mathrm{OD}}_{600\;\mathrm{nm}}\;\text{after vortexing})/\\ \left(\mathrm{OD}\;600\;\text{nmbefore vortexing}\right) \end{array}]\times 100\% \end{array}$$

#### 2.7.3 Inhibitory effect of Bak on bacterial biofilm formation

Bacterial suspensions of logarithmic growth phase and Bak (control, 1/4, 1/2, 1, 2, 4MIC) were co-cultivated in 96-well plates by the crystal violet method, and the total volume was adjusted to 200 μL and incubated at 37 °C for 12 h. The biofilm was stained with 0.1% crystal violet dye solution. Finally, the absorbance value of each well was detected at 600 nm and three replicate wells were set up in each treatment group for parallel determination (Ding et al., 2024).

#### 2.7.4 Effect on bacterial metabolism in biofilm

The bacterial suspension of logarithmic growth phase and Bak (control, 1/8, 1/4, 1/2, 1, 2, 4MIC) were co-cultured in 96-well plates by thiazolyl blue (MTT) method to make the final volume of 100 μL, and then incubated at 37 °C for 12 h. Then 20 μL of 5 mg/mL MTT was added, incubated at 37 °C for 4 h, and the OD was finally measured to be 570 nm. Each concentration was repeated in parallel 3 times (Sun et al., 2025).

### 2.8 Effects on the biofilm extracellular polymers

#### 2.8.1 Extracellular water-soluble/insoluble polysaccharides

Sulfuric acid-Anthrone method was use to determination of extracellular polysaccharide content (Wang et al., 2015). Prepared a series of concentration of glucose solution with reference to the ratio shown in Table 1. Finally, prepared anthrone reagent was added to each tube and plotted a standard glucose curve with glucose concentration (μg/mL) on the horizontal coordinate and OD_620nm_ values on the vertical coordinate and made three parallel controls for each concentration.

**Table 1:** Standard glucose content preparation table.

No.	0	1	2	3	4	5	6
Glucose standard solution /mL	0	0.2	0.4	0.6	0.8	1.0	1.2
Distilled water /mL	2.0	1.8	1.6	1.4	1.2	1.0	0.8
Glucose concentration/μg·mL^−1^	0	10	20	30	40	50	60

The bacterial solution, treated with different concentrations of Bak, was incubated at 37 °C for 24 h, followed by centrifugation at 2000 rpm for 15 min to collect the supernatant and the process was operated cyclically for three times. This wash cycle was performed three times, with the final supernatant intended for the assay of water-soluble extracellular polysaccharides. To the remaining bacterial pellet, added 5 mL of 0.2 mol/L sodium hydroxide solution. Centrifuged at 2000 rpm for 15 min, retained the supernatant, and repeated three times to determine the content of insoluble extracellular polysaccharides. Substituted the OD_620nm_ value of each sample into the linear regression equation of the standard glucose curve to calculate the release of water-soluble/insoluble extracellular polysaccharides.

#### 2.8.2 Extracellular proteins

The 50 mg/mL bovine serum albumin (BSA) standard solution was diluted to 1.0 mg/mL in PBS, and a protein quantification standard curve was prepared according to the Bradford assay (He et al., 2018). The logarithmic growth phase bacteria were incubated with different concentrations of Bak (control, 1/4MIC, 1/2MIC, MIC, 2MIC) at 37 °C for 4 h. This was followed by the addition of 4 mL of G250 staining solution and a 10-min incubation at room temperature. The absorbance at 595 nm was then measured spectrophotometrically, and the extracellular protein concentration was calculated accordingly.

#### 2.8.3 Extracellular eDNA

Add 3 mL of growth log phase bacterial solution and Bak of MIC to the 12-well plate, then add 1 mL of 6% milk solution, mix well, and incubate at 37 °C for 48 h. The one with BHI medium was used as a blank control, and the one with only bacterial solution was used as a negative control. Subsequently, 1 mL of 0.5 mol/L EDTA was added, and the mixture was left to stand at 4 °C for 1 h. The solution in the wells was then discarded, and 1 mL of 10 buffer (50 mmol/L) was added. The biofilm was fully resuspended by pipetting, and its absorbance was measured at 620 nm. The resulting suspension was centrifuged at 12,000 rpm for 10 min. From the supernatant, 500 μL was collected and mixed with chloroform: isoamyl alcohol (24:1) by vortexing for 30 s. The sample was washed three times with 70% anhydrous ethanol. The pellet was then dissolved in 10 μL of TE buffer to release extracellular DNA (eDNA). The eDNA concentration was determined by measuring the absorbance at 260 nm and 280 nm. The ratio of OD_260/_OD_280_ was calculated, and the eDNA content per unit of biofilm was quantified based on these measurements (Rice et al., 2007).

### 2.9 Untargeted metabolomic analysis

#### 2.9.1 Sample preparation and processing

1% (v/v) bacterial culture in the logarithmic growth phase was inoculated into BHI medium and incubated at 37 °C with shaking until the early logarithmic phase. The culture was treated with Bak at 3.12 μg/mL (MIC) for 8 h, with an equal volume of DMSO as a blank control. Each group included three independent biological replicates (*n* = 3). After centrifugation, the bacterial pellet was flash-frozen in liquid nitrogen for storage.

Frozen samples were thawed at 4 °C. A 100 mg aliquot was placed in a centrifuge tube, and 1 mL of extraction solvent (water, acetonitrile, and isopropanol in a 1:1:1 ratio) was added. The mixture was vortexed for 1 min and sonicated for 30 min at low temperature. After centrifugation at 12000 rpm for 10 min at 4 °C, the supernatant was frozen at −20 °C for 1 h to precipitate proteins. The sample was centrifuged again under the same conditions, and the clear liquid was vacuum-dried. The residue was reconstituted in 200 μL of 30% acetonitrile aqueous solution, vortexed, centrifuged, and the clear liquid was used for analysis.

#### 2.9.2 LC–MS detection and analysis

1. Liquid chromatography conditions

A Waters HSS T3 column (100 × 2.1 mm, 1.8 μm particle size) was used, with 0.1% formic acid in water as mobile phase A and 0.1% formic acid in acetonitrile as mobile phase B. The flow rate was 0.3 mL/min, the column temperature was 40 °C, and the injection volume was 2 μL. The gradient program was as follows: initial 100% A for 1 min, linear increase to 95% B over 12 min, hold for 1 min, then return to 100% A and maintain until 17 min.

1. Mass spectrometry conditions

Data were acquired using a Thermo Q Exactive HFX high-resolution mass spectrometer equipped with an electrospray ionization (ESI) source. Parameters were set as follows: sheath gas flow rate, 40 units; auxiliary gas flow rate, 10 units; spray voltage, +3,000 V (positive mode) or −2,800 V (negative mode); ion source temperature, 350 °C; capillary temperature, 320 °C. The instrument operated in full scan-data-dependent MS2 (Full-MS-ddMS2) mode with alternating positive and negative ion detection. The mass range for MS1 was 70–1,050 Da, with a resolution of 70,000 for MS1 and 17,500 for MS2. QC samples were prepared by pooling equal aliquots of all test samples and injected periodically throughout the acquisition sequence. The relative standard deviation (RSD) of metabolite peak areas was calculated to evaluate instrument stability.

#### 2.9.3 Data preprocessing

Initial data processing was performed using Progenesis QI (Waters, Milford, USA), including baseline correction, peak detection, alignment, retention time adjustment, and peak matching, to generate a data matrix of retention time, m/z, and peak area. Metabolites were identified based on commercial databases and an in-house secondary mass spectral library, with structural annotation guided by characteristic fragmentation patterns. Identification reliability was quantified by MS2 matching scores (Score, range 0–1), where scores closer to 1 indicated higher confidence (Score > 0.5 was considered reliable). Differential metabolites were identified by combining fold change (FC) and Benjamini–Hochberg corrected *p* values. The screening thresholds were FC > 1.5 or FC < 0.67 and *p* < 0.05.

### 2.10 Acute toxicity in mice

All animals used in the acute toxicity study were supplied by the Laboratory Animal Center of Lanzhou University, and the study protocol was approved by the institutional ethics review committee (Approval License Numbers: SCXK2023-0003). The median lethal dose (LD_50_) was determined using the modified Karber method (Li et al., 2025; Xu et al., 2024). Animals received a single oral gavage at a dose of 4 mg/kg; if adverse reactions were observed, another group received 1 mg/kg. Other groups were administered Bak at doses of 1426.93 mg/kg, 2011.97 mg/kg, and 2836.88 mg/kg. The control group received the same volume of vehicle without any compound. Following administration, the animals underwent multiple clinical examinations daily for seven consecutive days. If animals exhibit any signs of pain or distress, including marked immobility, respiratory distress, hypothermia, convulsions or coma, they shall be euthanized via carbon dioxide inhalation. Each group consists of six mice, with three replicates per group.

### 2.11 Validation of efficacy on mouse mastitis model

#### 2.11.1 Preparation of bacterial suspension

Acute mammary gland model of mice was established with *S. agalactiae* takedown. The optimal concentration of *S. agalactiae* tapping was first determined by pre-experimentation using plate colony counting method.

#### 2.11.2 Establishment of mouse mastitis model and group sampling

All animal experiments were conducted in compliance with National Institutes of Health Guidelines for the Care and Use of Laboratory Animals. All procedures were approved by the Ethics Committee of Lanzhou University’s (Approval License Numbers: SCXK2023-0003). Female mice at peak lactation were selected and randomly divided into five groups: blank control group, negative control group, positive control group, Bak high dose group (150 mg/kg), Bak low dose group (50 mg/kg), five mice in each group. A total of 50 μL of bacterial suspension at the optimal concentration (10^6^ CFU/mL) was injected into the mammary duct of the fourth mammary gland of mice, while the blank control group received an equal volume of normal saline. Twenty four hours later, the blank and negative control groups were intragastrically administered normal saline; the positive control group was given norfloxacin (50 mg/kg) by gavage. For the treatment groups, the gavage volume was calculated at 0.2 mL/10 g body weight according to the corresponding dose and body weight of each mouse. The drug was administered at a fixed time every day for 7 days. On the eighth day after drug treatment, mice were anesthetized by a single intraperitoneal injection of pentobarbital sodium at 40 mg/kg. The bilateral fourth pair of mammary glands were harvested upon dissection. One gland was fixed in 4% paraformaldehyde in phosphate-buffered saline, and the other was stored at −80 °C. The mice were subsequently euthanized via carbon dioxide inhalation (Che et al., 2023).

#### 2.11.3 Observation of mouse mammary gland pathological tissue sections

Fixed mouse mammary pathological tissues were dehydrated and embedded in paraffin, sectioned at 4 μm, then deparaffinized and rehydrated through a graded ethanol series. The sections were stained with hematoxylin–eosin (HE), dehydrated and cleared in ethanol and xylene, cover slipped, and examined and imaged by light microscopy (Wang et al., 2025).

#### 2.11.4 RT-PCR to detect the relative expression of mRNA of inflammatory factors

Mammary tissue RNA extraction was performed according to RNA simple Total RNA Extraction Kit. RNA was reverse transcribed to cDNA, and the reaction system and reaction procedure of the samples were the same as 2.7. The relative expression levels of target mRNAs were quantified using the 2^−ΔΔCt^ method. The primer sequences are shown in Table 2.

**Table 2:** RT-qPCR upstream and downstream primers.

Gene	Upstream primer (5′-3′)	Downstream primer (5′-3′)
TNF-α	GGTGCCTATGTCTCAGCCTCTT	GCCATAGAACTGATGAGAGGGAG
IL-1β	TGGACCTTCCAGGATGAGGACA	GTTCATCTCGGAGCCTGTAGTG
IL-6	CTGCAAGAGACTTCCATCCAG	AGTGGTATAGACAGGTCTGTTGG
β-actin	GGCTGTATTCCCCTCCATCG	CCAGTTGGTAACAATGCCATGT

### 2.12 Statistics and analysis

The experimental data were presented in the form of “mean ± standard deviation”, and one-way analysis of variance (ANOVA) combined with Duncan’s multiple testing method was used for comparison between groups (*p* < 0.05). Statistical operations were performed by SPSS 26.0 statistical software.

## 3 Results and discussion

### 3.1 Antibacterial activity and structure–activity relationship

The activity of 24 small-molecule phenolic compounds against four Gram-positive bacteria was determined, as shown in Table 3. Results indicated that most compounds exhibited no antibacterial activity against the four Gram-positive bacteria at a concentration of 100 μg/mL. Among them, Olivetol, Gallic acid isoamyl ester, Magnolol, Honokiol, Bavachin, Corylifolinin, and Bak demonstrated excellent antibacterial activity, particularly against *S. agalactiae*, *S. dysgalactiae,* and *S. aureus*. Further antimicrobial activity assays revealed that Bak exhibited a MIC of 3.12 μg/mL against *S. agalactiae*, *S. dysgalactiae,* and *S. aureus*, while Corylifolinin showed MIC values of 3.12 μg/mL, 3.12 μg/mL, and 6.25 μg/mL against *S. agalactiae*, *S. dysgalactiae,* and *S. aureus*, respectively (As was shown in Table 4). Notably, both Bak and Corylifolinin contained an isoprenyl moiety, indicating that isopreny group enhanced antimicrobial activity.

**Table 3:** Antibacterial inhibition of major secondary metabolites (%).

Name	Structure	Inhibition rate (%) ± standard deviation (*n* = 3)			
		GBS	*S.d*	*E.c*	*S.a*
Carvacrol	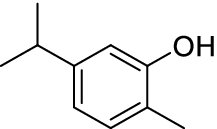	0.00 ± 0.00	40.90 ± 4.26	0.00 ± 0.00	6.54 ± 2.20
Thymol	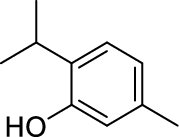	30.21 ± 4.18	58.72 ± 3.07	0.00 ± 0.00	53.58 ± 1.33
Olivetol	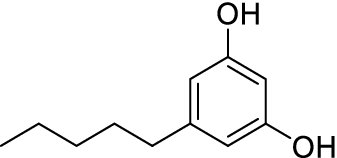	96.51 ± 0.68	100.00 ± 0.00	68.29 ± 1.77	81.62 ± 0.55
Gallic acid	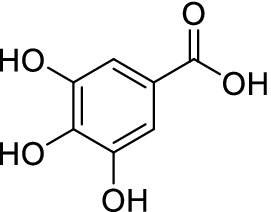	75.64 ± 1.60	0.00 ± 0.00	0.00 ± 0.00	31.74 ± 2.78
Methyl gallate	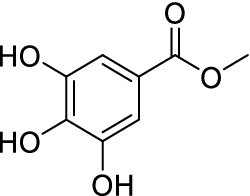	39.46 ± 3.05	26.39 ± 3.16	0.00 ± 0.00	21.67 ± 1.63
Ethyl gallate	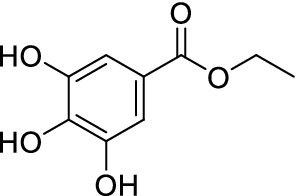	41.52 ± 0.87	69.91 ± 2.33	0.00 ± 0.00	32.61 ± 0.80
Butyl gallate	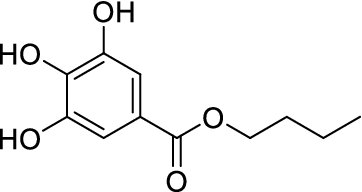	43.28 ± 3.06	67.44 ± 4.64	22.30 ± 1.79	53.81 ± 0.71
Gallic acid isoamyl ester	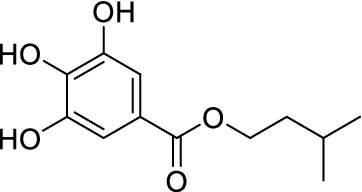	84.01 ± 5.42	99.00 ± 0.83	2.61 ± 0.80	73.73 ± 0.49
Protocatechuic acid	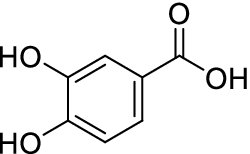	0.00 ± 0.00	40.67 ± 0.00	0.00 ± 0.00	23.74 ± 5.17
Vanillin	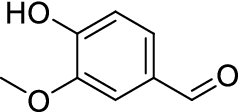	10.92 ± 0.86	45.90 ± 2.42	0.00 ± 0.00	16.08 ± 3.82
trans-Ferulic acid	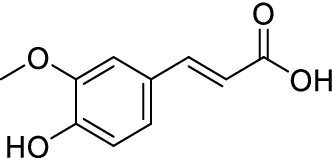	33.33 ± 5.94	27.4 ± 4.05	9.62 ± 0.83	52.13 ± 0.35
Ethyl 4-hydroxy-3-methoxycinnamate	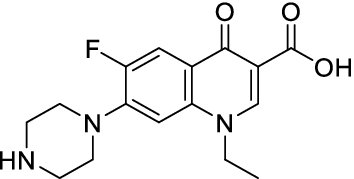	36.78 ± 1.61	18.42 ± 1.17	0.00 ± 0.00	33.00 ± 2.16
2-Allylphenol	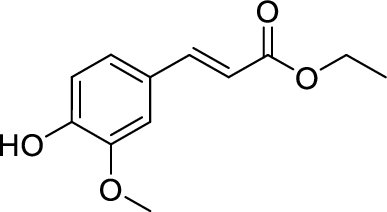	0.00 ± 0.00	8.98 ± 0.73	0.00 ± 0.00	0.00 ± 0.00
Estragole	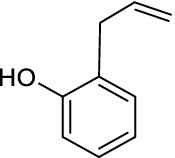	5.90 ± 1.97	0.00 ± 0.00	0.00 ± 0.00	7.52 ± 1.46
trans-Anethole	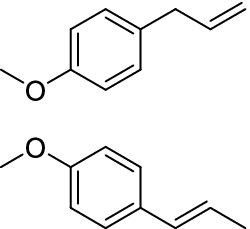	12.26 ± 0.35	0.00 ± 0.00	0.00 ± 0.00	0.00 ± 0.00
Eugenol	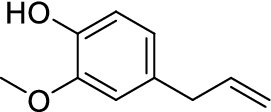	0.00 ± 0.00	0.00 ± 0.00	8.35 ± 1.29	27.35 ± 4.01
Methyl eugenol	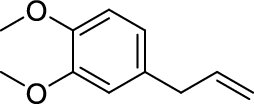	4.69 ± 1.54	0.00 ± 0.00	0.00 ± 0.00	38.21 ± 5.69
Isoeugenol	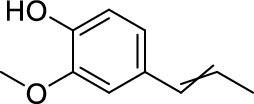	0.00 ± 0.00	0.00 ± 0.00	0.00 ± 0.00	0.00 ± 0.00
Methyl isoeugenol	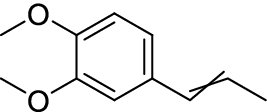	0.00 ± 0.00	0.00 ± 0.00	0.00 ± 0.00	0.00 ± 0.00
Magnolol	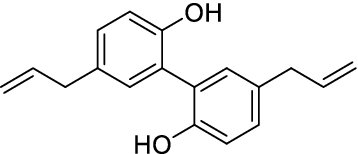	100.00 ± 0.00	99.65 ± 0.47	0.00 ± 0.00	99.21 ± 1.31
Honokiol	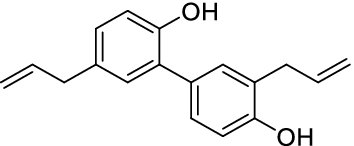	100.00 ± 0.00	100.00 ± 0.00	6.24 ± 0.12	99.74 ± 0.26
Bakuchiol	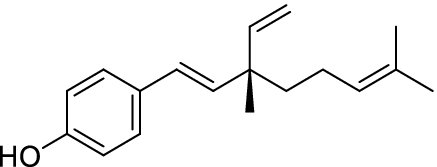	98.64 ± 1.32	99.30 ± 2.42	0.00 ± 0.00	100.00 ± 0.00
Bavachin	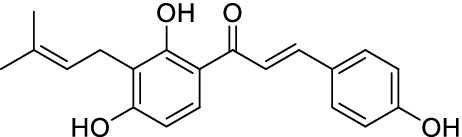	100.00 ± 0.00	98.11 ± 1.96	0.00 ± 0.00	100.00 ± 0.00
Corylifolinin	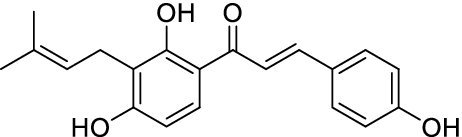	100.00 ± 0.00	98.11 ± 1.96	0.00 ± 0.00	100.00 ± 0.00
Norfloxacin	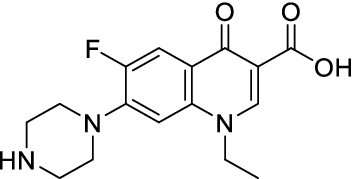	100.00 ± 0.00	98.84 ± 0.00	100.00 ± 0.00	100.00 ± 0.00

**Table 4:** MIC (μg/mL) of highly active compounds against mastitis-causing bacteria in dairy cows.

Name	Inhibition rate (%) ± standard deviation (n = 3)			
	GBS^a^	*S.d*	*E.c*	*S.a*
Olivetol	100	100	>100	>100
Bakuchiol	3.12	3.12	>100	3.12
Gallic acid isoamyl ester	>100	100	>100	>100
Magnolol	50	50	>100	12.5
Honokiol	25	12.5	>100	6.25
Bavachin	50	>100	>100	50
Corylifolinin	3.12	3.12	>100	6.25
Norfloxacin	12.5	6.25	<0.39	0.78

^a^GBS, *Streptococcus agalactiae* ATCC 27956; S. d, *Streptococcus dysgalactiae* ATCC 27957; S. a, *Staphylococcus aureus Newman*; E. c, *Escherichia coli* ATCC 25922.

Based on the above results, structure–activity relationship analysis of 24 small-molecule phenolic compounds revealed that Olivetol exhibited superior antibacterial activity compared to Carvacrol and Thymol, indicating that introducing hydroxyl groups and saturated carbon side chains enhanced antibacterial activity. Compared to Gallic acid, Methyl gallate, Ethyl gallate, and Butyl gallate, Gallic acid isoamyl ester exhibited significantly increased activity, suggesting that longer carbon chains correlated with higher activity. Studies on 2-Allylphenol, Estragole, Eugenol, Methyl eugenol, and Isoeugenol demonstrated that altering the position of the alkene on the benzene ring or replacing the hydroxyl group with a methoxy group did not enhance activity. Comparing magnolol and honokiol activity, we found that biphenyl structures enhanced antimicrobial activity, but ortho-positioned hydroxyl groups exhibited greater activity than para-positioned ones. Compared to Bak, isoprenyl groups increased compound activity, with longer side chains yielding better results. According to these findings, we selected the most active compound-Bak for mechanism studies.

### 3.2 Antibacterial activity of Bak

The growth curve evaluated the inhibitory effect of Bak on *S. agalactiae*. As shown in Figure 1, *S. agalactiae* entered the logarithmic phase after 2 h of incubation, grew rapidly, and stabilized after 10 h. However, treatment with a concentration of 1/2 MIC delayed entry into the logarithmic growth phase in *S. agalactiae*, indicating a certain degree of inhibitory effect. When the concentration was increased to MIC and 2MIC, Bak completely inhibited the growth of *S. agalactiae*, demonstrating that Bak has strong, concentration-dependent antibacterial activity against this organism (see Table 5).

**Figure 1:**
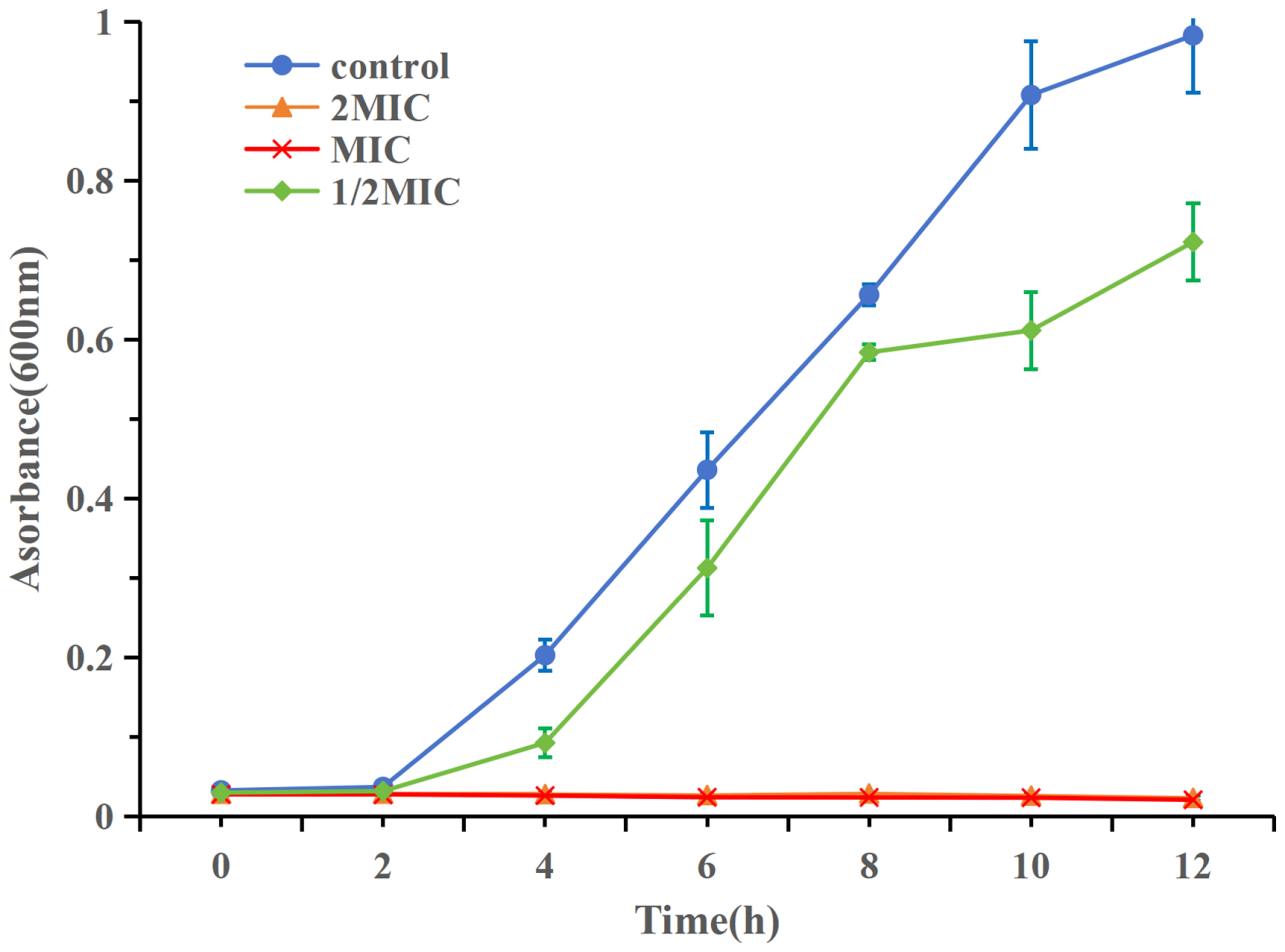
Effect of different concentrations of Bak on growth curves of *S. agalactiae*.

**Table 5:** Experimental results of hemolytic inhibition rate.

Group	Logistic fitting equation	R^2^	EC_50_(cfu/mL)	HT	IR
Control	y = 80.640–162.849/(1 + (x/4.426E7)^0.941)	0.981	4.426E7	22.593	78.04%
Bak	y = 106.070–110.291/(1 + (x/2.015E8)^1.931)	0.999	2.015E8	4.962	

### 3.3 Effects of Bak on cellular micromorphology

SEM observations visually proved the antimicrobial effect of Bak. As shown in Figures 2A,B, the untreated *S. agalactiae* cells maintained a regular, full shape and smooth surface, whereas the surface of the Bak-treated organisms appeared to be concave and even broken and deformed, indicating that the confirmed compounds caused irreversible damage to the bacteria and disrupted the structural integrity of the cells.

**Figure 2:**
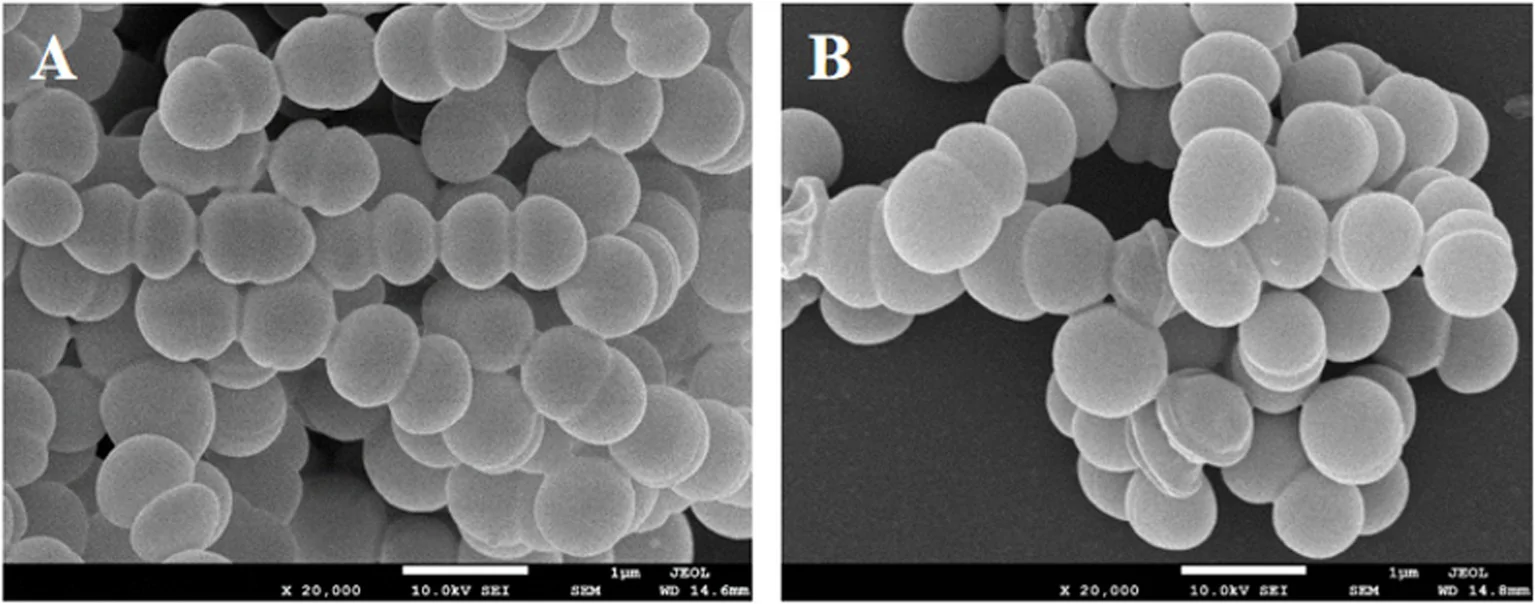
SEM images of *S. agalactiae* before and after Bak treatment. **(A)** Control **(B)** MIC concentration of Bak treatment. The scale bar was 1 μm.

### 3.4 Effects on virulence factors of Bak

*β*-Hemolysin is a pore-forming toxin encoded by the *CylE* gene, which has lysogenic properties that allow *S. agalactiae* to lysate host cells, resulting in disruption of cellular structure and function (Xin et al., 2024). The effect of Bak on *β*-hemolysin production was quantified and analyzed using a two-fold gradient dilution method. The results showed that 3.12 μg/mL of Bak treated *S. agalactiae*, according to Figures 3A,B fitting equations [y = 80.640–162.849/(1 + (x/4.426E7)^0.941), R^2^ = 0.981] yielded the hemolytic rate of *S. agalactiae* with the concentration of EC_50_ of 4.426 × 10^7^ CFU/mL, and the HT of 22.593, the IR of 78.04%, indicating that Bak exhibited a strong inhibitory ability to show hemolysin.

**Figure 3:**
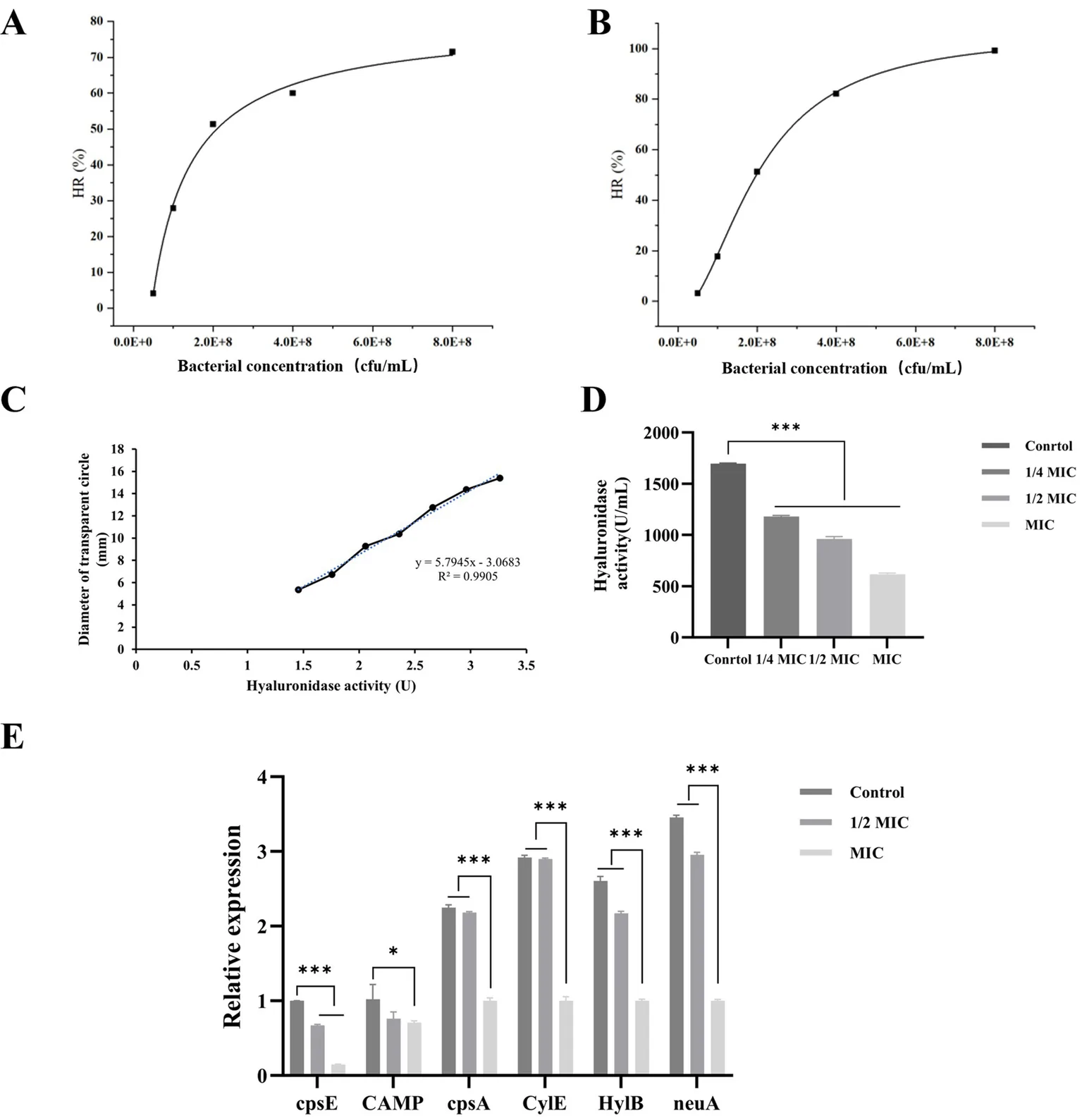
Effect of Bak on the virulence of *S. agalactiae*. Effect plots of the Logisitic equation fitted to the bacterial concentration and hemolysis rate in the control group **(A)** and the MIC Bak-treated group **(B)**. **(C)** Standard curve for hyaluronidase. **(D)** Effect on hyaluronidase activity. **(E)** Effect on expression of virulence genes.

The standard curve of hyaluronidase (Figure 3C) fitting equation was measured as y = 5.7945lg(x)−3.0683 (R^2^ = 0.9905). Compared to the blank control (Figure 3D), the hyaluronidase inhibition rates were 30.37%, 43.31%, and 63.67% at 1/4MIC, 1/2MIC, and MIC, respectively. Thus, compared to the control group, lower concentrations of Bak could inhibit hyaluronidase production. As Bak concentration increases, the inhibitory effect gradually strengthened, indicating that Bak could suppress hyaluronidase production in a concentration-dependent manner.

The results of virulence gene expression experiments are shown in Figure 3E. Compared to the control group, after Bak treatment, the expression of virulence genes related to invasion and immune evasion, including hemolysin (*CylE*), CAMP factor (CAMP), hyaluronidase (*HylB*), capsular polysaccharide-associated virulence factors (*CpsA*, *CpsE*), and the enzyme responsible for O-acetylation of capsular polysaccharide (*neuA*), were significantly down-regulate and the higher the concentration, the lower the gene expression level (*p* < 0.05).

### 3.5 Effects on biofilms

The first stage of biofilm formation is both the bacterial adhesion phase, during which the bacteria attach to the host cell surface and coalesce, which is necessary for the biofilm formed by the bacteria. As shown in Figure 4A, at a concentration of 1/2 MIC, Bak slightly inhibited the initial attachment of *S. agalactiae*, reducing the adhesion rate by 6.51%. When the concentration increased to MIC, the adhesion rate decreased by 36.32%. At a concentration of 2 MIC, the inhibition of bacterial adhesion became more pronounced, with the adhesion rate reduced by 75.27%. These results indicated that Bak effectively interfered with the bacterial attachment process during the early stages of biofilm formation, significantly weakening the ability to form biofilms.

**Figure 4:**
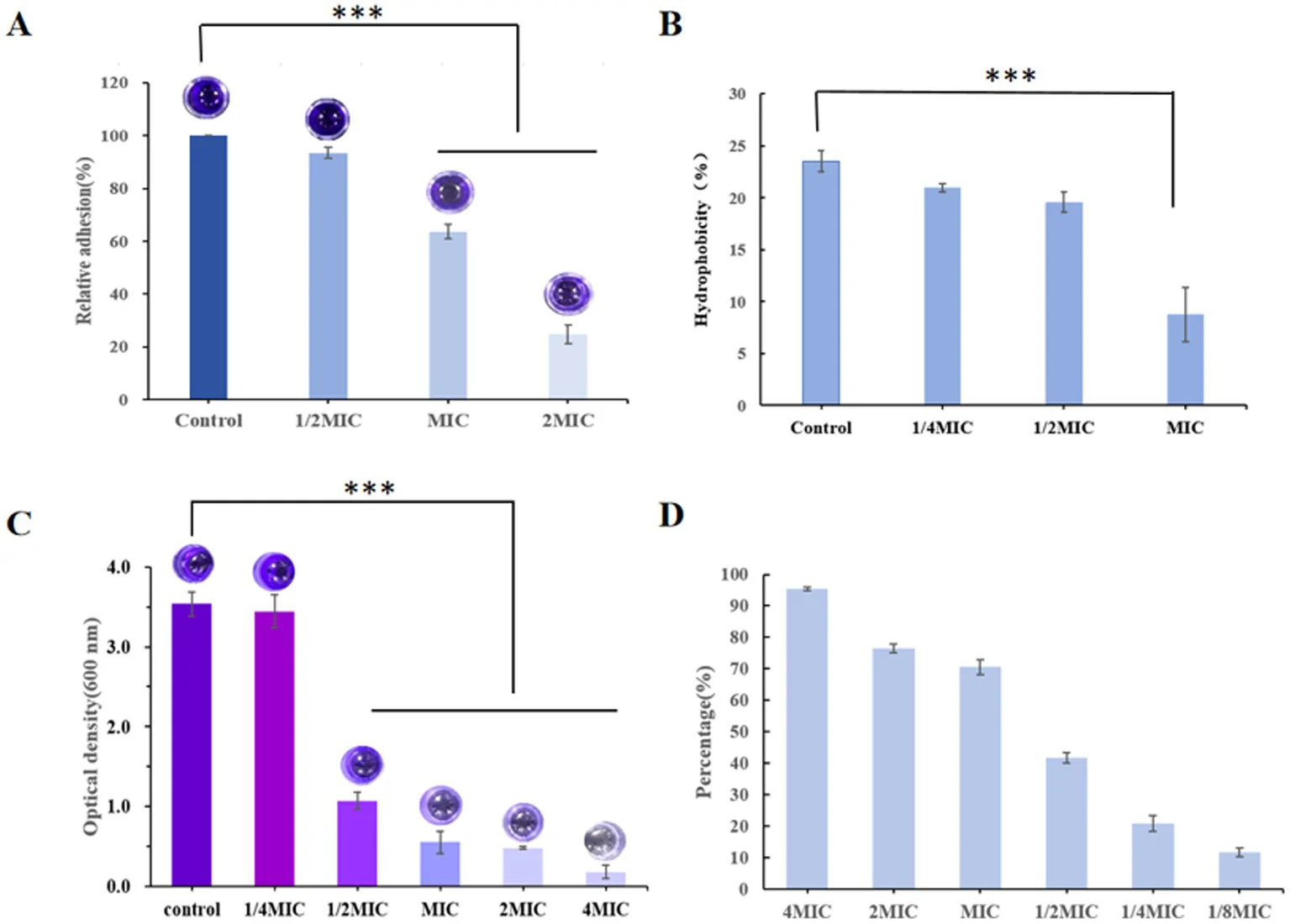
Effect of Bak on *S. agalactiae* biofilms. **(A)** Adhesion experiments in the early stages of biofilm formation. **(B)** Effect on cellular hydrophobicity in the early stages of biofilm formation; **(C)** Scavenging effect on mature biofilms. **(D)** Effect on bacterial metabolism within biofilms.

Cell surface hydrophobicity is the basis of bacterial adhesion, biofilm formation and disease causing, which makes the adhesion and agglutination of cells increased and contributes to the continuous expansion of bacterial colonies. The experimental results are shown in Figure 4B. The surface hydrophobicity of bacterial biofilm decreased by 58.3% after Bak treatment at MIC concentration. The results indicated that Bak action decreased the cell surface hydrophobicity in a concentration-dependent manner and reduced the biofilm formation ability.

As shown in Figure 4C, Bak inhibited bacterial biofilm formation at different concentrations ranging from 1/4 ~ 4 MIC with biofilm removal rates of 2.73%, 69.67%, 84.40%, 86.33 and 94.80%, respectively. In addition, the effect of the corresponding crystal violet staining at each concentration can be clearly seen, with decreasing staining intensity according to the concentration from low to high. This study reveals that Bak has a significant scavenging effect on mature biofilms.

Bacterial cell viability was assessed using the MTT assay to evaluate its interference with biofilm metabolic functions (Samen et al., 2007). As shown in Figure 4D, at a concentration of 1/8 MIC, Bak exhibited a 11.75% inhibition rate against membrane metabolic activity, indicating a weak inhibitory effect. At concentrations of 1/4 MIC and 1/2 MIC, the inhibition rates were 20.87% and 41.79%, respectively. As the concentration increased to MIC and 2MIC, the inhibition rates reached 70.55% and 76.54%, respectively, indicating significant inhibition. At a concentration of 4MIC, Bak inhibited membrane metabolism by 95.47%, nearly completely suppressing metabolic activity.

### 3.6 Effects on biofilm extracellular polymers

Extracellular polysaccharides (EPS) are extracellular carbohydrate polymers that form capsules or mucus layers around cell. EPS assists in bacterial cell attachment, protects the cell from harsh environmental conditions, desiccation, and antimicrobial agents, and aids in bacterial survival, acclimatization, continued colonization, and biotic and abiotic stress tolerance (Kaur and Dey, 2023). The experimental results showed (Figures 5A–C) that the linear regression equation of the standard glucose curve y = 0.071 + 0.012x (*R*^2^ = 0.995), indicating a linear relationship between glucose content and absorbance. The results showed that the addition of Bak all decreased the content of water-soluble/insoluble polysaccharides. For water-soluble polysaccharides the inhibition was 11.54% at 1/2MIC concentration and 40.16% at MIC concentration; for water-insoluble polysaccharides the inhibition was 5.94% at 1/2MIC concentration and 87.30% at MIC concentration.

**Figure 5:**
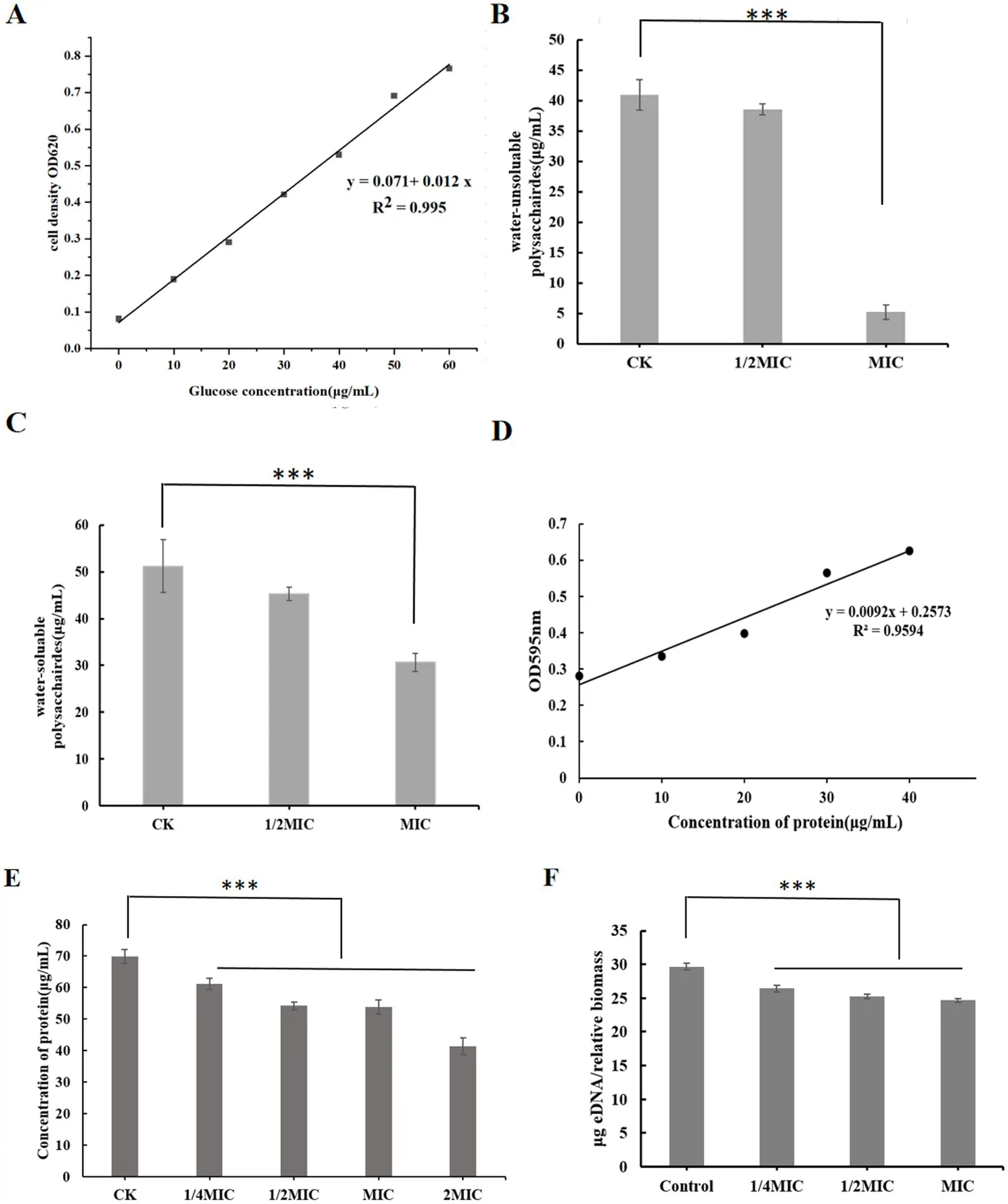
Effect of Bak on extracellular polymers of biofilm. **(A)** Glucose standard curve. **(B)** Extracellular water-insoluble polysaccharide content. **(C)** Extracellular water-soluble polysaccharide content. **(D)** Protein standard curve. **(E)** Extracellular protein content. **(F)** Extracellular eDNA content.

Extracellular proteins are also one of the key components of the extracellular polymers of the biofilm, and they also play an important role in the normal biological function of the biofilm. As shown in Figure 5D, the standard curve equation of the protein is y = 0.0092 x + 0.2573 (R^2^ = 0.9594). In accordance with the standard curve of protein, the amount of extracellular protein in the bacterial broth of each mixed culture was converted. As shown in Figure 5E, compared with the control group, all concentrations of Bak had a certain inhibitory effect on extracellular proteins, especially after the effect of 2MIC of Bak, the content of extracellular proteins decreased by 40.66%, which indicates that Bak can inhibit the synthesis and secretion of extracellular proteins.

Extracellular DNA (eDNA) contributes to bacterial adhesion, metal chelation, metabolic viability, antibiotic resistance, and horizontal gene transfer, and it is key to bacterial adhesion and aggregation, especially in the early stages of biofilm formation (Panlilio and Rice, 2021). The effect of Bak on eDNA is shown in Figure 5F. eDNA content of biofilm eDNA changed significantly after treatment with different concentrations of Bak, and the decrease of eDNA was more pronounced with the increase of the concentration, compared with the control group, after MIC Bak treatment.

### 3.7 Untargeted metabolomics analysis

#### 3.7.1 Analysis of differential metabolites

In this study, samples were separated and identified using high-performance liquid chromatography-mass spectrometry. In both positive and negative ion modes, the identified metabolites were statistically grouped into 11 major categories, as shown in Figure 6A. Different substance categories are represented by distinct colored regions, with percentages indicating each category’s proportion among all metabolites. Major categories include Carboxylic acids and derivatives (31.5%), Organooxygen compounds (8.65%), Fatty Acyls (8.07%), and other compounds (32%).

**Figure 6:**
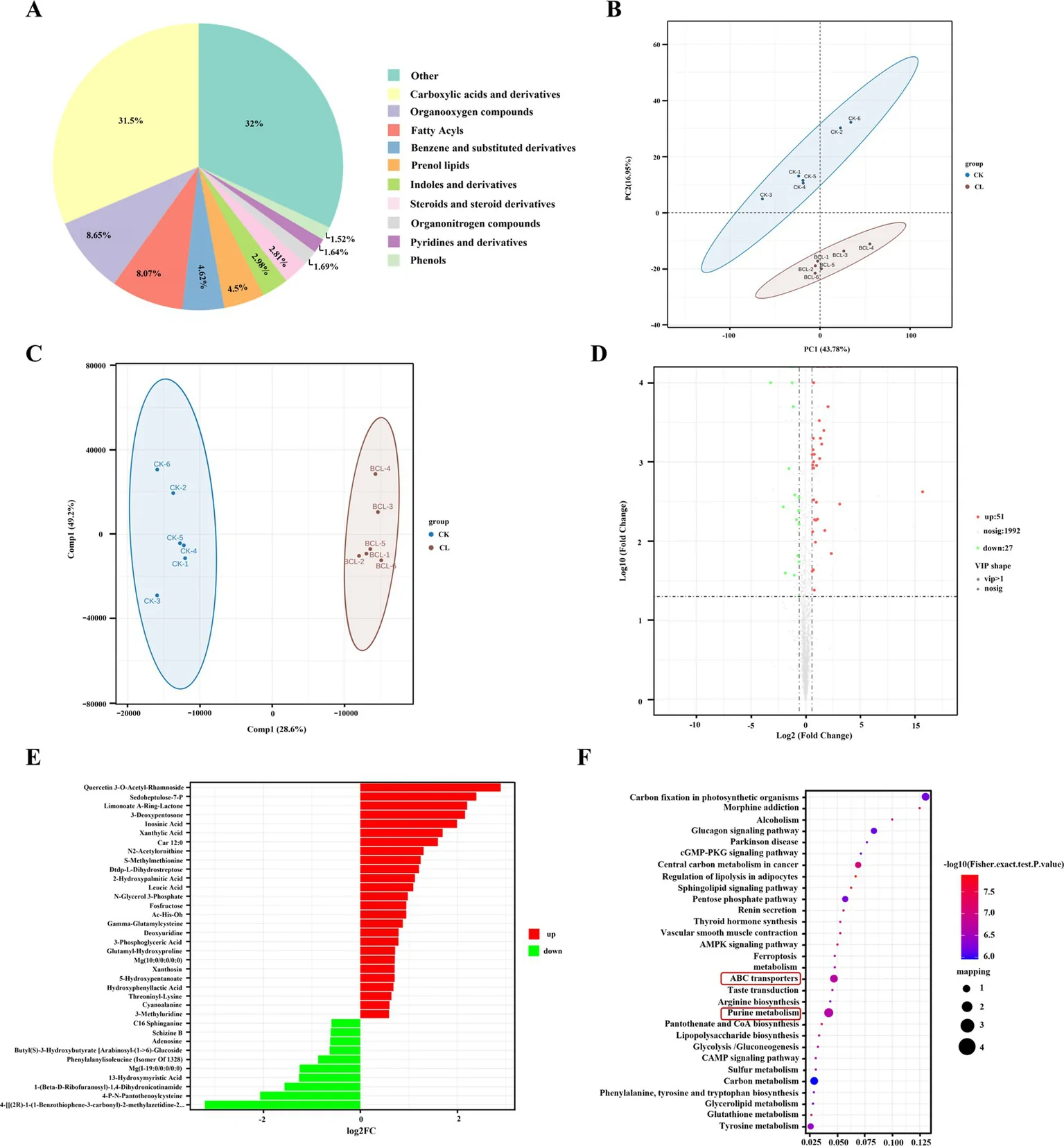
Untargeted metabolomics analysis of the *S. agalactiae*-treated group. **(A)** Metabolite pie plot. **(B)**Score scatter plot of PCA model for group treated and control. **(C)** Scatter plot of OPLS-DA scores for group treated and control. **(D)** Volano plot of differential metabolites between treated and control group. **(E)** Bar chart of differential metabolite FC for between treated and control groups. **(F)** KEGG metabolic pathway bubble chart for between treated and control groups.

Principal component analysis (PCA) is a statistical method. By performing PCA analysis on all bacterial metabolites from the Bak-treated group and the control group, we can understand the overall differences between the sample groups and the degree of variation within each group. As shown in Figure 6B, whether in positive ion detection mode or negative ion detection mode, the samples treated with Bak and the control group exhibited overlapping patterns while demonstrating clear distinguishability. This indicates significant differences between the metabolite profiles of the two groups, confirming that Bak treatment induced substantial alterations in the overall metabolic levels of *S. agalactiae*. Orthogonal Projections to Latent Structures-Discriminant Analysis (OPLS-DA) was employed for further intergroup metabolite differential analysis as shown in Figure 6C. The samples from the treatment and control groups were clearly distinguished, with all samples falling within the 95% confidence interval. This further indicates differences in the metabolome composition between the two groups. Moreover, in the OPLS-DA model, the R2Y and Q2 values for Bak relative to the control group were 0.99 and 0.94, respectively, demonstrating the high stability and reliability of the constructed model.

#### 3.7.2 Identification of differential metabolites

Based on the OPLS-DA model analysis, intergroup differential metabolites were screened using Variable Importance Projection (VIP) values. The screening criteria were set as FC > 1.5 or FC < 0.67 and *p* value < 0.05, VIP > 1. As shown in Figure 6D, 78 significantly differentially expressed metabolites were identified, comprising 51 significantly upregulated metabolites and 27 significantly downregulated metabolites. Statistical analysis of the metabolites with the greatest fold change among the differentially expressed metabolites revealed significant upregulation of Quercetin 3-O-Acetyl-Rhamnoside, Sedoheptulose-7-P, Inosinic Acid, Xanthylic Acid, 3-Deoxypentosone, and Fosfructose were significantly upregulated, while 1-(Beta-D-Ribofuranosyl)-1,4-Dihydronicotinamide, Mg(I-19:0/0:0/0:0), 13-Hydroxymyristic Acid, 4-P-N-Pantothenoylcysteine, and C16 Sphinganine were significantly downregulated (Figure 6E).

#### 3.7.3 KEGG enrichment analysis

KEGG pathway enrichment analysis employs Fisher’s Exact Test to assess the significance level of metabolite enrichment across pathways, thereby identifying significantly affected metabolic and signaling pathways. In the enrichment results, a smaller *p*-value (*p* < 0.05) indicates statistically more significant KEGG pathway enrichment. As shown in Figure 6F, the metabolites produced by Bak treatment are primarily concentrated in the ABC transporter and purine metabolism pathways. In microbial metabolism, purine metabolism constitutes one of the major components of energy metabolism, and purine nucleotides are also essential constituents of nucleic acids and cofactors (Bønsdorff et al., 2004; Schwechheimer et al., 2018). Disruption of purine metabolism impairs ATP synthesis, hindering the energy supply required for bacterial amino acid synthesis (Goncheva et al., 2022). This reduces the production of virulence factors such as extracellular proteins and β-hemolysin, weakens bacterial metabolism within biofilms, and impairs cell membrane synthesis and extracellular polymer formation. ABC transporters are a class of proteins that transport various essential molecules-such as monosaccharides, amino acids, lipids, polysaccharides, and proteins-across cell membranes via ATP hydrolysis (Ao et al., 2025; Wang H. et al., 2022b). Research indicates that ABC transporter-dependent pathways participate in the biosynthesis of bacterial extracellular polysaccharides, suggesting that Bak may influence extracellular polysaccharide production through ABC transport pathways, thereby inhibiting biofilm synthesis (Khan et al., 2020; Lansky et al., 2020). Additionally, ABC transporters are involved in the synthesis of cell surface proteins. Research indicates that most bacterial surface proteins are secreted via the Type II secretion pathway (How et al., 2025), suggesting Bak may influence cell surface protein synthesis through the ABC transporter pathway. This could disrupt bacterial adhesion, leading to impaired cell hydrophobicity and inhibition of mature cell membrane formation. Furthermore, quorum sensing plays a crucial role in bacterial resistance, including regulating extracellular matrix secretion and biofilm formation. This suggests Bak may inhibit extracellular matrix secretion via quorum sensing (Bhargava et al., 2010).

### 3.8 Toxicity evaluation of Bak

As shown in Table 6, the mortality of mice reached 100% at the Bak dose of 4,000 mg/kg. When the concentration was reduced to 2836.88 mg/kg, the mortality rate decreased to 83.33% after 7 days. As the concentration of Bak gradually decreased, its mortality rate progressively declined. At a concentration of 1012.01 mg/kg, the mortality rate among mice was 0%, with a survival rate of 100%. Based on the above results, the LD_50_ of Bak in mice was calculated to be 1897.6 mg/kg, with a 95% confidence interval of 1564.7 to 2306.6 mg/kg. According to the World Health Organization’s classification criteria for oral toxicity of exogenous substances, Bak was considered a low-toxicity compound (500–5,000 mg/kg).

**Table 6:** Acute toxicity experiment results.

Groups	Dose(mg/kg)	Number of animals	Number of dead animals	Mortality rate (%)	Survival rate (%)
1	1012.01	6	0	0.00	100.00
2	1426.93	6	1	16.67	83.33
3	2011.97	6	2	33.33	66.67
4	2836.88	6	5	83.33	16.67
5	4000.00	6	6	100.00	0.00

### 3.9 Validation of the efficacy of Bak in a model of mouse mastitis

#### 3.9.1 Establishment and observation of mouse mastitis model

In this experiment, after injecting 50 μL of a 10^6^ CFU/mL concentration into the fourth mammary duct, the mice exhibited reduced vitality but did not die. Twenty-four hours later, mice in the negative control group began exhibiting symptoms such as depression, ruffled fur, abdominal swelling, breast hardening, darkened nipples, and mammary tissue detachment, as shown in Figure 7A, along with severe congestion. In contrast, observation of the positive control and treatment groups revealed gradual recovery in mental state, food intake, and water consumption during feeding. Post-mortem examination showed reduced hemorrhagic foci and lighter coloration in mammary tissue (as shown in Figures 7B–D). Surprisingly, the high-dose group (150 mg/kg) demonstrated superior therapeutic effects compared to the low-dose group (50 mg/kg), with results approaching those of the positive control. Compared with the blank control group, both the positive control group and the Bak treatment group showed significantly reduced disease severity and superior recovery outcomes. Additionally, the high-dose treatment group exhibited recovery patterns essentially identical to the positive control group, while its therapeutic efficacy was inferior to that of the low-dose treatment group.

**Figure 7:**
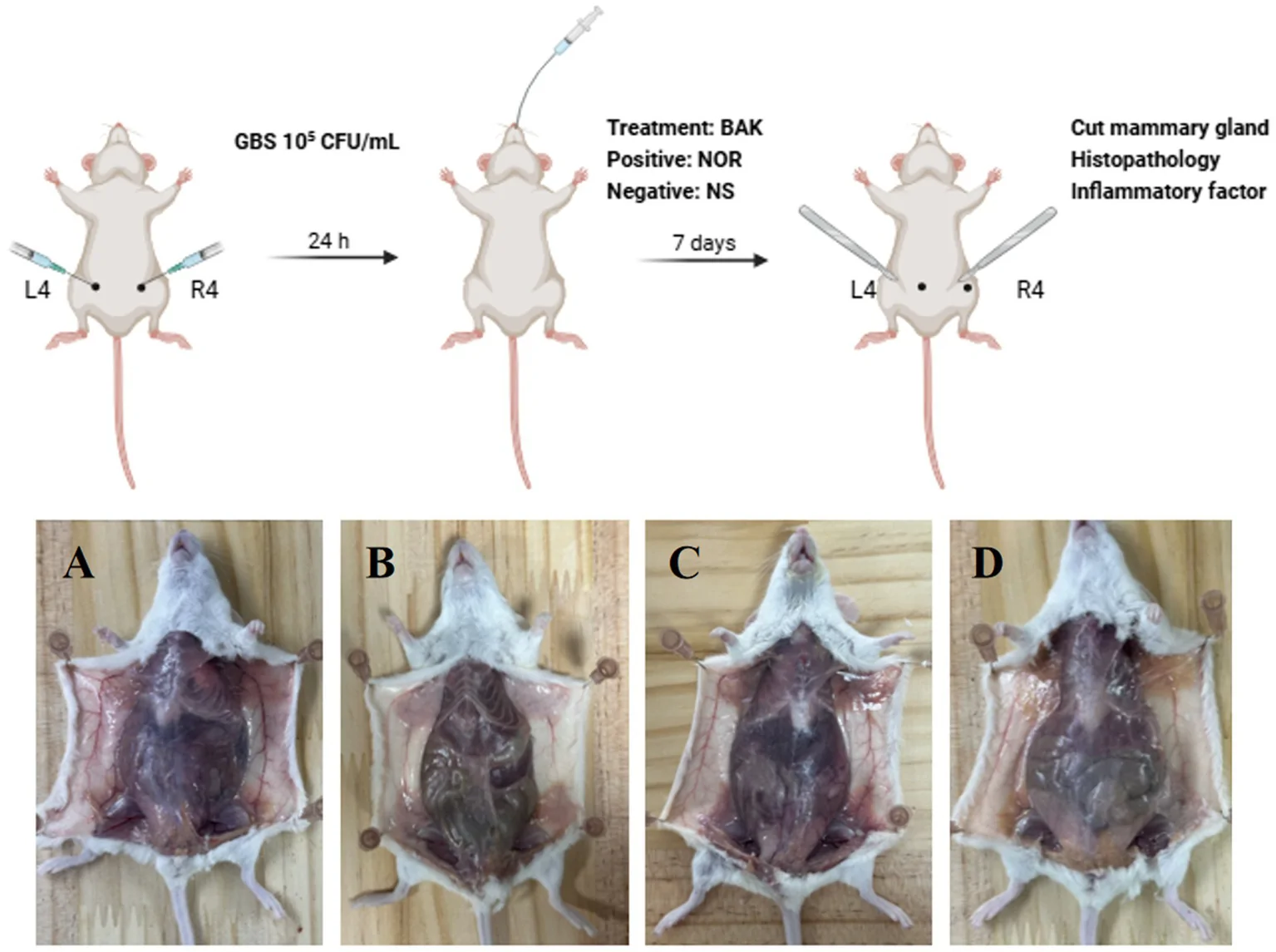
Modeling of mouse mastitis (generated by BioRender.com) and observation of mammary tissue. **(A)** Negative control group (*S. agalactiae* infection model control). **(B)** Positive control (50 mg/kg norfloxacin treatment group). **(C)** Low dose group (50 mg/kg) Bak treatment group. **(D)** High dose group (150 mg/kg) Bak treatment group.

#### 3.9.2 Observations on pathological tissue sections of mouse mammary gland

The results of HE histopathology showed that in the negative control group (Figure 8A), severe pathological changes were seen, with a lot of acinar morphology changes, narrowing of the acinar lumen, more epithelial cells with loose cytoplasm (gray arrows), and a few fragments of necrotic cells were seen in the acinar lumens.

**Figure 8:**
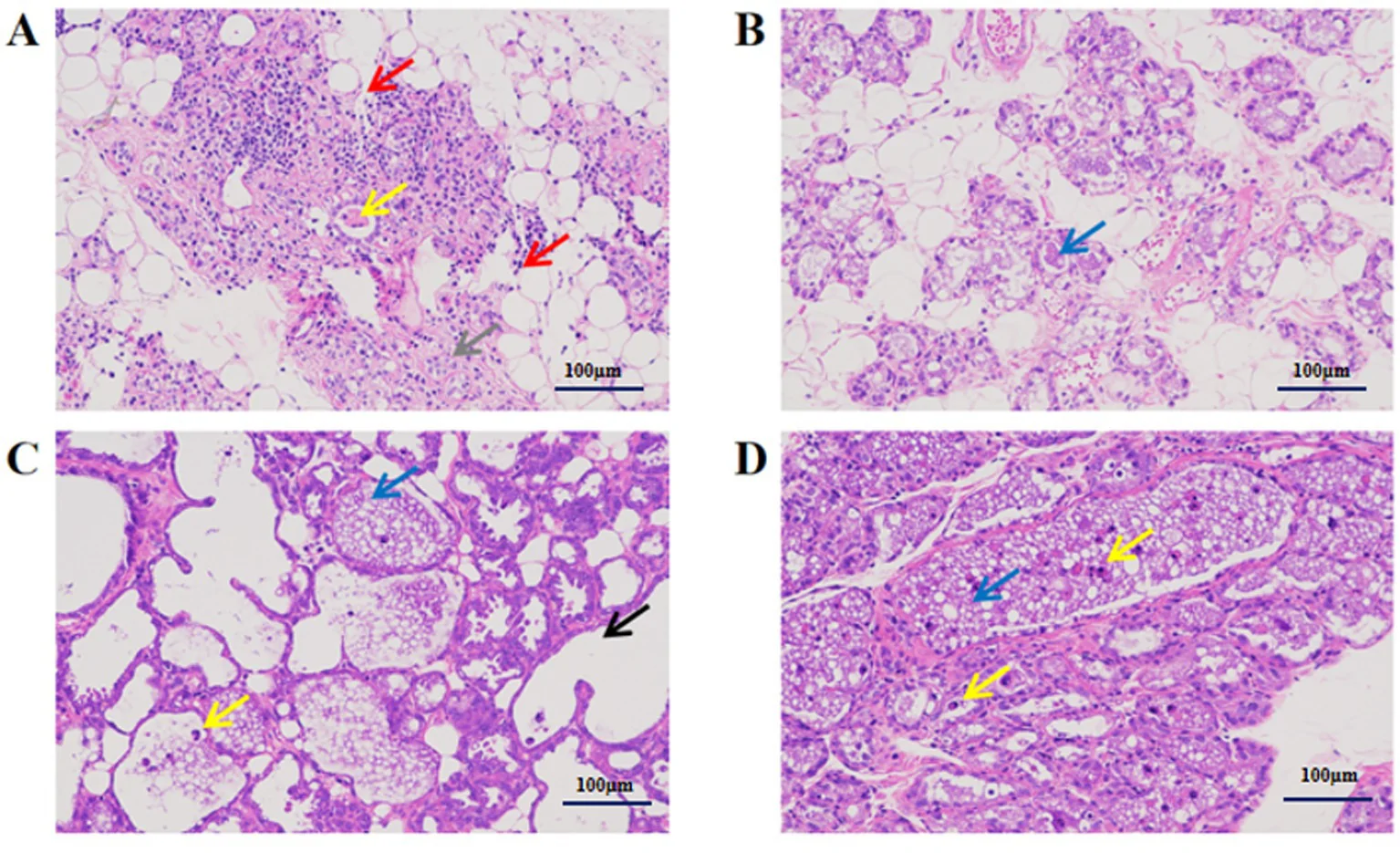
Observation of pathologic tissue sections. **(A)** Negative control group. **(B)** Positive control. **(C)** Low-dose group (50 mg/kg) Bak treatment group. **(D)** High-dose group (150 mg/kg) Bak treatment group. Yellow arrows represent necrotic cell debris and blue arrows represent eosinophilic material.

A large amount of adipose tissue was seen in the interstitium, and a large number of lymphocytes and granulocytes were seen in scattered infiltration (red arrows). The positive control group (Figure 8B) had a high number of acinar cells with no obvious pathologic changes except for the presence of a small amount of eosinophilic material. In the low-dose treatment group (Figure 8C) a small number of acinar cells were disrupted, but acinar dilatation was seen (black arrows), as well as a large amount of eosinophilic material with necrotic cellular debris. In the high-dose treatment group (Figure 8D), the number of acinar cells was large and tightly arranged, and a small amount of eosinophilic material with necrotic cell debris was visible, which was reduced relative to the blank and low-dose groups.

#### 3.9.3 Relative expression of inflammatory factor mRNA in mouse mammary tissue

As shown in Figure 9, the mRNA expression of pro-inflammatory-related cytokines IL-6, IL-1β, and TNF-α in mouse mammary tissues was significantly increased in the Blank control group (CG group), compared with Negative control group (BG) (*p* < 0.01). Compared with CG, Bak treatment groups (B-1, B-2) significantly reduced the mRNA expression levels of these pro-inflammatory-related cytokines (*p* < 0.01). At the molecular level, Bak was able to alleviate the inflammatory response of breast tissue induced by *S. agalactiae* infection.

**Figure 9:**
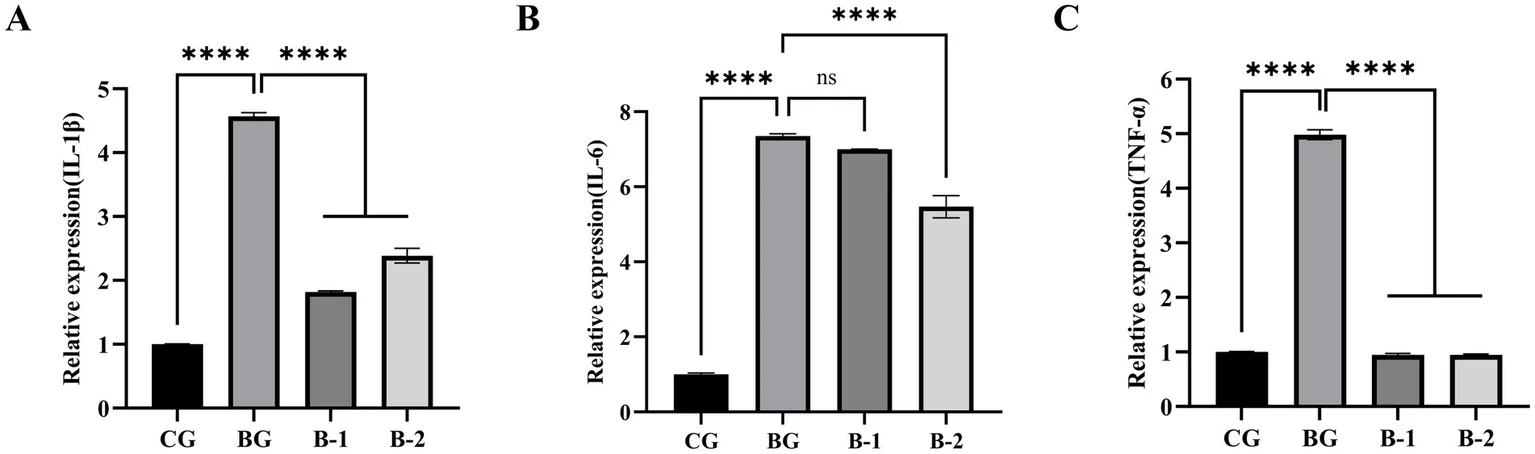
**(A)** IL-1β. **(B)** IL-6. **(C)** TNF-α. (CG) Negative control group. (BG) Blank control group. (B-1) Low dose treatment group. (B-2) High dose treatment group.

In summary, this study demonstrated that Bak exhibited significant activity against *S. agalactiae*. It exerted its antibacterial effects through multiple pathways of interaction. Bak disrupted the morphology of *S. agalactiae* cells, reduced the activity of β-hemolysin and hyaluronidase, and inhibited the expression of virulence factors. Furthermore, Bak inhibited biofilm formation, affected adhesion, cell hydrophobicity, and removal of mature biofilms, while reducing metabolic activity in biofilm-forming bacteria. Concurrently, Bak diminished secretion of biofilm-associated virulence factors such as extracellular polysaccharides, extracellular proteins, and extracellular eDNA. Furthermore, Untargeted metabolomics revealed that Bak might exerted its effects by influencing purine metabolism and ABC transporter pathways, thereby affecting bacterial ATP production and ABC transporter transport mechanisms. In a mouse mastitis model, Bak demonstrated significant therapeutic efficacy by reducing tissue damage and downregulating the expression of inflammatory cytokines (IL-6, IL-1β, TNF-α). Importantly, acute toxicity studies in mice revealed Bak was a low-toxicity compound. Therefore, this study provided a highly promising lead compound for the prevention and treatment of *S. agalactiae* infections.

## Data Availability

The raw data supporting the conclusions of this article will be made available by the authors, without undue reservation.
